# Acupuncture Treatment for Chronic Pelvic Pain in Women: A Systematic Review and Meta-Analysis of Randomized Controlled Trials

**DOI:** 10.1155/2018/9415897

**Published:** 2018-09-27

**Authors:** Soo-Hyun Sung, Angela-Dong-Min Sung, Hyun-Kyung Sung, Tteul-E-Bom An, Kyeong Han Kim, Jang-Kyung Park

**Affiliations:** ^1^Department of Pathology, College of Korean Medicine, Dae-gu Haany University, Daegu 38610, Republic of Korea; ^2^Department of Preventive Medicine, College of Korean Medicine, Sangji University, Wonju 26339, Republic of Korea; ^3^Department of Korean Pediatrics, College of Korean Medicine, Semyung University, Jechon 27136, Republic of Korea; ^4^Department of Obstetrics and Gynecology, College of Korean Medicine, Dae-gu Haany University, Daegu 38610, Republic of Korea; ^5^Department of Preventive Medicine, College of Korean Medicine, Woosuk University, Wanju 55338, Republic of Korea; ^6^Department of Obstetrics and Gynecology, College of Korean Medicine, Sangji University, Wonju 26339, Republic of Korea

## Abstract

**Aim of the Study:**

This systematic review and meta-analysis aims to evaluate the current evidence from randomized controlled trials (RCTs) related to the effectiveness and safety of acupuncture treatment (AT), including electroacupuncture or thread-embedding therapy in combination with modern technology, for chronic pelvic pain (CPP) in women.

**Materials and Methods:**

We searched 12 electronic databases up to December 2017. All randomized controlled trials evaluating the effect of AT for CPP were considered.

**Results:**

Four RCTs with 474 participants were included. The methodological quality of included studies was generally low. The results of meta-analysis of two studies showed that AT combined with conventional treatment (CT) was associated with significantly reduced CPP, based on the total effectiveness rate (n=277, mean difference = 1.29, confidence interval = 1.13 to 1.47, P=0.0001, I^2^ = 0%).

**Conclusions:**

This review suggests the potential of AT combined with CT compared to CT alone for treating female CPP. However, there is insufficient evidence to conclude that AT can be recommended as a complementary and alternative (CAM) treatment for women with CPP. To draw a firm conclusion, future studies should require not only lager, more rigorously designed RCTs but also research on different AT types.

**Protocol Registration Number:**

This study is registered with PROSPERO 2018 (CRD42018088627).

## 1. Introduction

Chronic pelvic pain (CPP) is noncyclic pain of more than 6 months that localizes in the pelvis, the anterior abdominal wall at or below the umbilicus, the lumbosacral region of the spine, or the buttocks [1, 2]. A total of 14.7% of women aged 18-50 years in the United States experience CPP within the prior three months [3]. Severe CPP not only causes functional disability in patients, but also reduces quality of life [1, 4].

Although there is no clear understanding of the mechanism of CPP, the European Association of Urology (EAU) guidelines suggest that inflammation or infection of somatic or visceral tissue, central nervous system (CNS) activity, and emotional, cognitive, behavioural, and sexual components are involved [5]. The guidelines include a description of the diagnosis and treatment of CPP according to a predefined mechanism [5].

In traditional Korean medicine, the main causes of CPP are thought to be static blood or depression of seven emotions and are treated with acupuncture or herbal medicine [6, 7].

Acupuncture has long been used and is effective in relieving pain and is also minimally invasive, inexpensive, and safe [8, 9].

Acupuncture used in combination with modern technology, for example, electroacupuncture (EA), delivers electrical current through acupuncture, and acupoint thread-embedding therapy (TET) maximizes stimulation by inserting thread into meridian points [10].

Recently, comprehensive guidelines for the diagnosis and treatment of CPP have been developed by the EAU [5]. However, there is no evidence-based complementary and alternative medicine (CAM) treatment. Moreover, no published systematic review has determined whether acupuncture treatment (AT) (e.g., classic acupuncture, EA, and TET) for CPP is safe and effective.

This systematic review and meta-analysis aims to evaluate current evidence from randomized controlled trials (RCTs) to assess the effectiveness and safety of AT for CPP.

## 2. Methods

### 2.1. Protocol and Registration

This systematic review was registered in the PROSPERO 2018 (available from http://www.crd.york.ac.uk/PROSPERO/display_record.php?ID=CRD42018088627).

### 2.2. Data Sources and Searches

The following electronic databases were searched to identify relevant studies for inclusion in the review from inception to December 2017: the Cochrane Central Register of Controlled Trials (CENTRAL), MEDLINE, EMBASE, CINAHL Plus, two Chinese databases (the China National Knowledge Infrastructure (CNKI) and Wanfang), and six Korean databases (the National Digital Science Library (NDSL), the Korean Traditional Knowledge Portal, KoreaMed, the Oriental Medicine Advanced Searching Integrated System (OASIS), the Research Information Sharing Service (RISS), and The National Library of Korea).

The search terms used were (“chronic pelvic pain” OR “chronic pelycalgia” OR “chronic pain of pelvic” OR “chronic pelvic ache”) AND (“acupuncture” OR “acupoint” OR “needling” OR “electroacupuncture” OR “electro-acupuncture” OR “electric acupuncture” OR “hand acupuncture” OR “scalp acupuncture” OR “auricular acupuncture” OR “ear acupuncture”) AND (“Randomized controlled trial” OR “randomized clinical trial”).

### 2.3. Study Selection

All RCTs evaluating the effect of AT of CPP were included. Nonrandomized trials, animal or cell studies, and reviews were excluded. Women participants diagnosed with CPP were considered. Any type of AT (e.g., classic acupuncture, electroacupuncture, scalp acupuncture, auricular acupuncture, and thread-embedding therapy) for treating CPP was included. AT that does not involve the insertion of needles into the skin (e.g., acupoint pressure, or acupressure) was not considered. We included RCTs comparing AT with no treatment, placebo/sham treatment, or conventional treatment (CT). RCTs that assessed the combined effects of AT plus CT were also included when the identical CT was applied to both groups. Our primary outcome measure was the patient-reported pain score (e.g., visual analogue scale, numeric rating scale for CPP, or total effectiveness rate for CPP). As secondary outcomes, we examined quality of life, activity score, and adverse events (AEs).

### 2.4. Data Extraction

Two of the authors (A. D. Sung and H. K. Sung) independently reviewed and screened the titles and abstracts of the retrieved studies based on the predefined eligibility criteria. Two independent reviewer (S. H. Sung and T. E. An) extracted the following data from the included studies: author information, sample size, types of diseases, intervention and control groups, outcome measures, main results, and any adverse events. Any disagreements arising between the reviewers during this process were resolved through discussion with a third author (J. K. Park).

### 2.5. Assessment of Risk of Bias (ROB)

Two authors (S. H. Sung and K. H. Kim) independently evaluated the risk of bias of the included RCTs using the Cochrane Handbook V.5.1.0 [15]. This tool consists of seven domains, but we assessed the following six: random sequence generation, allocation concealment, blinding of participants and personnel, blinding of outcome assessments, incomplete outcome data, and selective reporting. For each domain, the risk of bias for each study was assessed according to three categories: low risk (L), high risk (H), or unclear (U). Disagreements encountered during the process were settled by a third author (J. K. Park) through discussion.

### 2.6. Data Analyses

For meta-analysis, we used RevMan software (Version 5.3.5 for windows; the Nordic Cochrane centre, Copenhagen, Denmark) [16]. Pooled dichotomous data were expressed as a risk ratio (RR) with 95% confidence interval (CI). In this case, we used a random-effects model for analysis and addressed heterogeneity among the included studies using the I^2^ test. I^2^ values above 50% or P values less than 0.10 showed considerable heterogeneity [11]. A summary of the findings was discussed in the results when a meta-analysis was not assessed.

## 3. Results

### 3.1. Study Selection and Description

**Figure 1:**
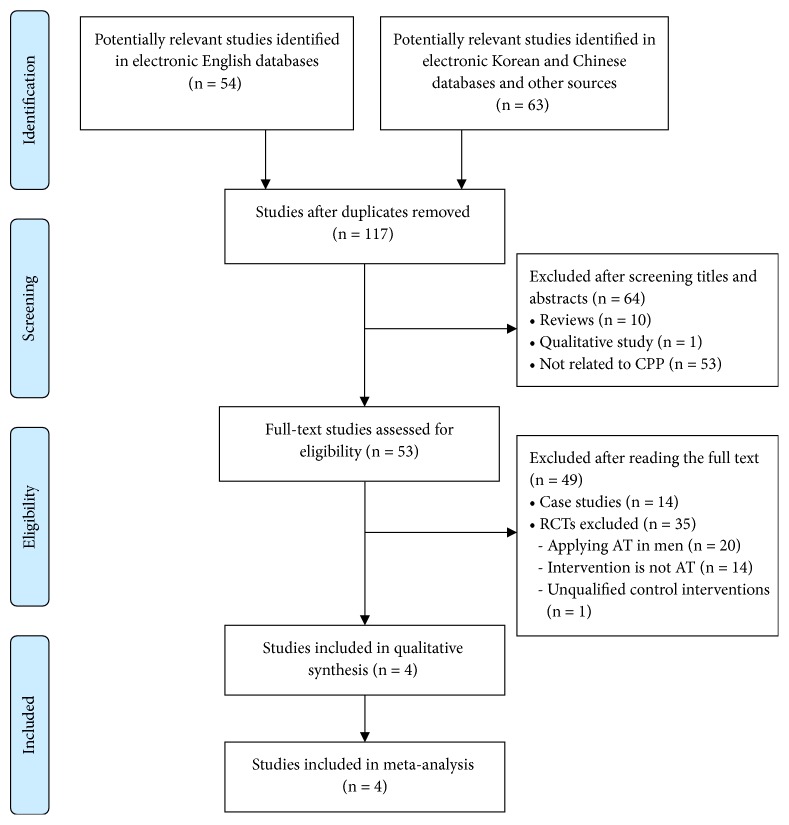
Flowchart of the RCT selection process. CPP: chronic pelvic pain; CCTs: controlled clinical trials; RCTs: randomized controlled trials; EAHM: external application of herbal medicine.

The searches identified 117 potentially relevant studies, of which 4 RCTs (English databases: n = 1; Chinese databases: n=3) met our inclusion criteria (Figure 1). Details of the included RCTs are summarized in Table 1. Three [12–14] of the 4 RCTs were conducted in China and published in Chinese. The remaining study [11] was conducted in Egypt and published in English.

### 3.2. Participants

A total of 474 CPP patients were included in the review. The number of participants was 250 in the experimental group and 224 in the control group. Three of the included RCTs assessed clinical conditions: pelvic adhesion [12] and pelvic inflammatory disease [13, 14].

### 3.3. Interventions

The types of AT in the RCTs varied: warm acupuncture with moxibustion on the handle of the needle was used in two studies [12, 13]; and EA [11] and TET combined with auricular acupuncture [14] were utilized in one study each.

Two of the included RCTs compared AT, including EA and TET combined with auricular acupuncture, with CT [11, 14]. Two other trials compared warm acupuncture plus CT with CT alone [12, 13].

### 3.4. Outcomes

#### 3.4.1. Acupuncture Treatment versus Conventional Treatment

Acupuncture treatment was compared with conventional treatment in two RCTs [11, 14], of which one contrasted EA with inferior hypogastric plexus blockade [11], while another compared TET plus auricular acupuncture with levofloxacin administration [14].

**Figure 2:**
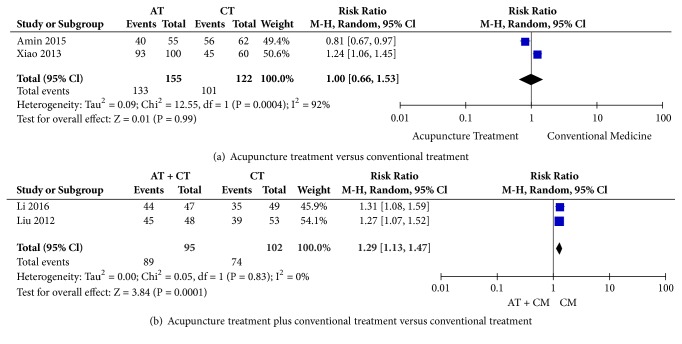
Meta-analysis of total effectiveness rate for chronic pelvic pain. AT: acupuncture treatment; CT: conventional treatment; CI: confidence intervals.

Two meta-analyses [11, 14] that compared the primary outcome of the total effectiveness rate (TER) for CPP between AT and conventional treatment showed no significant difference between the groups [Figure 2(a), mean difference (MD) = 1.00, confidence interval (CI) = 0.66 to 1.53, P = 0.99, I^2^ = 92%]. Amin [11] reported significant pain relief, measured with the visual analogue scale (VAS), in the AT group (P<0.001) and conventional treatment group (P<0.001).

#### 3.4.2. Acupuncture Treatment Plus Conventional Treatment versus Conventional Treatment

For the primary outcome of TER for CPP, data extracted from two RCTs [12, 13] showed significantly superior improvement in the experimental group compared to the control group [Figure 2(b), MD = 1.29, CI = 1.13 to 1.47, P=0.0001, I^2^ = 0%]. Li [12] reported significant efficacy based on the numeric rating scale (NRS) (P<0.05).

#### 3.4.3. Adverse Events

One RCT [11] reported that AEs did not occur.

### 3.5. Cochrane Risk of Bias Assessment

**Figure 3:**
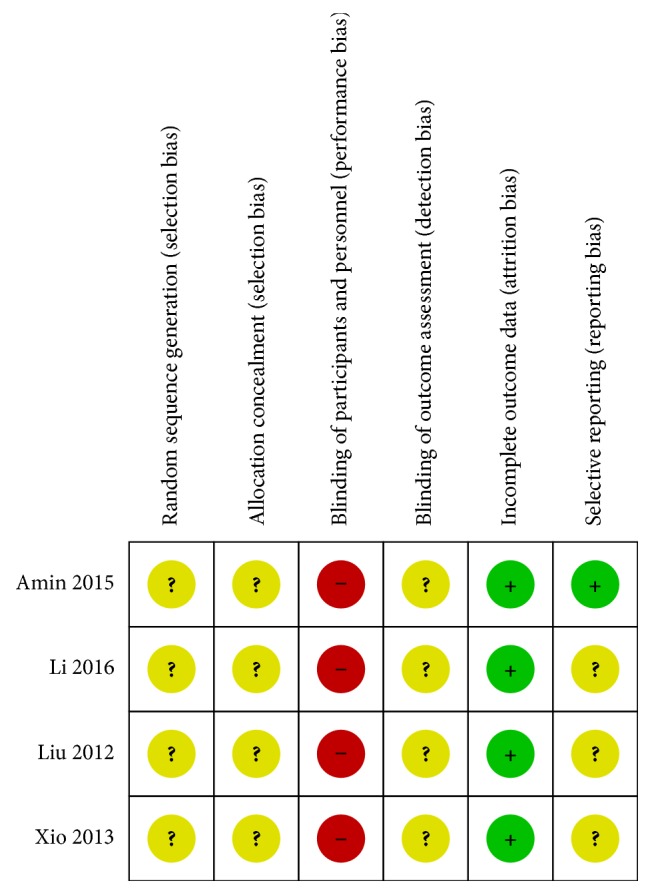
Summary risk of bias assessment.

The risk of bias of the included studies is presented in Figure 3. No included RCTs [8] mentioned the method of randomization or allocation concealment. Blinding of participants and practitioners was not performed in all of the included studies [8] due to differences in treatment type between groups. Moreover, these studies [8] did not report information about the blinding of the outcome assessors. All included RCTs [8] had a low risk of bias in addressing incomplete outcome data; three studies [8] had no missing outcome data; another trial [11] had missing outcome data, but the drop-out rate did not exceed 20% for short-term and 30% for long-term follow-up. In terms of selective reporting, only one [11] trial reported their protocol before conducting the studies.

## 4. Discussion

CPP in gynaecological practice is often associated with negative cognitive, behavioural, sexual, and emotional consequences and is often complex and difficult to treat [5]. Therapeutic options such as hormonal therapy or surgery are recommended in well-defined disease states and a multidisciplinary approach to pain management is used in persistent disease [5]. However in 30% of cases, no cause is ever determined and this presents a therapeutic challenge to the attending physician [5]. Thus, in the EAU guideline, the use of alternative therapies for chronic gynaecological pelvic pain is recommended [5].

Our systematic review provides suggestive evidence for the efficacy of AT, which is CAM therapy, in treating CPP. The meta-analysis that pooled data from two RCTs [12, 13] using the outcome measure of TER for CPP showed significant improvement with AT plus CT compared with CT alone (MD = 1.29, CI = 1.13 to 1.47, P=0.0001, I^2^ = 0%). However, the meta-analysis in two studies [11, 14] indicated that AT showed no significant improvement on the outcome of TER for CPP compared to that with CT (MD = 1.00, CI = 0.66 to 1.53, P = 0.99, I^2^ = 92%). Although our findings indicated that AT can be recommended as additional treatment when CPP patients are treated with CT, there is insufficient evidence to recommend evidence-based treatment with AT for female CPP due to heterogeneity of control interventions and the small number of trials in the included studies.

The strength of our review is that we searched various databases without language restriction to avoid publication bias. Thus, three East Asian RCTs were included in the review; the researchers assess Chinese language articles.

Our review has several limitations. First, most of the included studies had low methodological quality in the Cochrane ROB assessment. None of the studies provided information on generation of random allocation and the method of allocation concealment. The blinding of participants, practitioners, and outcome assessors was not performed in all included RCTs. Low methodological quality RCTs led to overestimation of treatment effects [17]. Furthermore, three [12–14] of 4 studies did not provide a published protocol or register it prior to execution. Registration of clinical trial protocols is important to identify whether a trial is affected by selective or incomplete outcome reporting [18]. Future studies should be registered in an open-accessible registry such as ClinicalTrials.gov. or WHO.int/ICTRP [19, 20].

Second, in terms of safety, only one RCT [11] reported that AEs did not occur in 117 CPP patients. Safety is a fundamental principle in medical treatment. In general, there is a common impression that acupuncture is safer and is therefore recommended as alternative treatment. Recent research reported that minor and rare serious AEs can occur during acupuncture [21]. Therefore, AEs must be reported in RCTs of CPP in the future to draw firm conclusions on the severity and frequency of AEs due to AT.

Third, our findings were limited due to variation of AT types; three types of AT, including warm acupuncture, EA, and TET were included in the review. Traditionally, dry acupuncture needles have been used, and as new types of AT in combination with modern science and technology have been developed and utilized, the range of treatment tools is expanding. Thus, clinical studies according to different types of AT should be investigated in the future.

Lastly, the primary outcome of TER for CPP used in meta-analysis is not an internationally accepted tool for measuring pain. TER, an outcome measure generally used in China, was graded according to the following categories: clinical cure, markedly effective, effective, and ineffective [22]. The validity and reliability of TER have not yet been verified. In the future, internationally recognized measurements such as VAS or NRS should be used.

Although this review presented the applicability of AT for female CPP patients, standardization of AT intervention was not examined. Therefore, studies should consider the following factors: (1) AT type; (2) duration of treatment and number of treatment sessions based on each AT type; (3) size and depth of needle; (4) acupuncture points; and (5) appropriate placebo model for each AT type. Researchers need to investigate efficacy and safety of AT in CPP to establish CAM treatment guidelines that reflect our findings.

## 5. Conclusion

The results of our review and meta-analysis suggest the effectiveness of AT combined with CT for treating women with CPP compared to use of CT alone. However, current evidence is insufficient to verify the efficacy of AT for CPP because of the small number of RCTs and low methodological quality and heterogeneity of interventions. Therefore, larger, more rigorous and adequately powered multicentre RCTs are needed to provide clinical guidelines for AT in treating female CPP patients.

## Figures and Tables

**Table 1:** Characteristics of the included RCTs for pelvic pain.

First author, year	Type of conditions related to pelvic pain	sample size (randomized//analysed)	Experimental group (intervention, regimen)	Control group (intervention, regimen)	Outcome measures	Main results	AEs
Amin, 2015 [11]	n.r.	127/117	(A) Electro-acupuncture, n=55, 12 sessions (2 times per week for 6 weeks)	(B) CT (inferior hypogastric plexus blockade), n=62, 1 sessions	(1) VAS (2) TER for chronic pelvic pain	(1) Significant difference in (A)^c^ and (B)^c^ (2) (B) better than (A)	None

Li, 2016[12]	Pelvic adhesion	96/96	(A) WA (using moxibustion) + CT (physical therapy), n=47, 10 sessions (1 times per day for 10 days)	(B) CT (physical therapy), n=49, 10 sessions (1 times per day for 10 days)	(1) NRS (2) TER for chronic pelvic pain	(1) Positive^a^ (2) Positive^a^	n.r.

Liu, 2012 [13]	Pelvic inflammatory disease	101/101	(A) WA (using moxibustion) + CT (cefuroxime axetil), n=48, 7 sessions for WAT (1 times per day for 7 days) and 14 sessions for CM (2 times per day for 7 days)	(B) CT (cefuroxime axetil), n=53, 14 sessions (2 times per day for 7 days)	(1) TER for chronic pelvic pain	(1) Positive^b^	n.r.

Xiao, 2013[14]	Pelvic inflammatory disease	160/160	(A) TET + AA, n=100, 2 sessions for TEF (1 times per 4 weeks for 8 weeks) and 8 sessions for AA (1 times per week for 8 weeks)	(B) CT (levofloxacin capsule), n=60, 2 sessions (2 times for week)	(1) TER for chronic pelvic pain	(1) Positive^a^	n.r.

^a^p < 0.05; ^b^p < 0.01; ^c^p < 0.001

AA: auricular acupuncture; AEs: adverse events; CT: conventional treatment; n.r.: not reported; NRS: Numerical Rating Scale; NS: no significant difference; QOL: quality of life; TET: thread embedding therapy; VAS: visual analogue scale; WA: warm acupuncture.
